# Supplementary material for: Species-specific host factors rather than virus-intrinsic virulence determine primate lentiviral pathogenicity
Source: Nat Commun. 2018 Apr 10;9:1371. doi: 10.1038/s41467-018-03762-3 (PMC5893559; doi:10.1038/s41467-018-03762-3)
Supplement: Supplementary file 1 — Supplementary Information(PDF 2325 kb) [file 41467_2018_3762_MOESM1_ESM.pdf]

SUPPLEMENTARY INFORMATION

Species-specific host factors rather than virus-intrinsic virulence determine primate lentiviral pathogenicity

Simone Joas,[1] Erica H. Parrish,[2$] Clement W. Gnanadurai,[1#] Edina Lump,[1] Christina M. Stürzel,[1] Nicholas F. Parrish,[2%] Gerald H. Learn,[2] Ulrike Sauermann,[3] Berit Neumann,[3] Kerstin Mätz Rensing,[3] Dietmar Fuchs,[4] James M. Billingsley,[5] Steven E. Bosinger,[5] Guido Silvestri,[5] Cristian Apetrei,[6] Nicolas Huot,[7,8] Thalia Garcia-Tellez,[7] Michaela Müller-Trutwin,[7] Dominik Hotter,[1] Daniel Sauter,[1] Christiane Stahl-Hennig,[3] Beatrice H. Hahn,[2] and Frank Kirchhoff[1*]

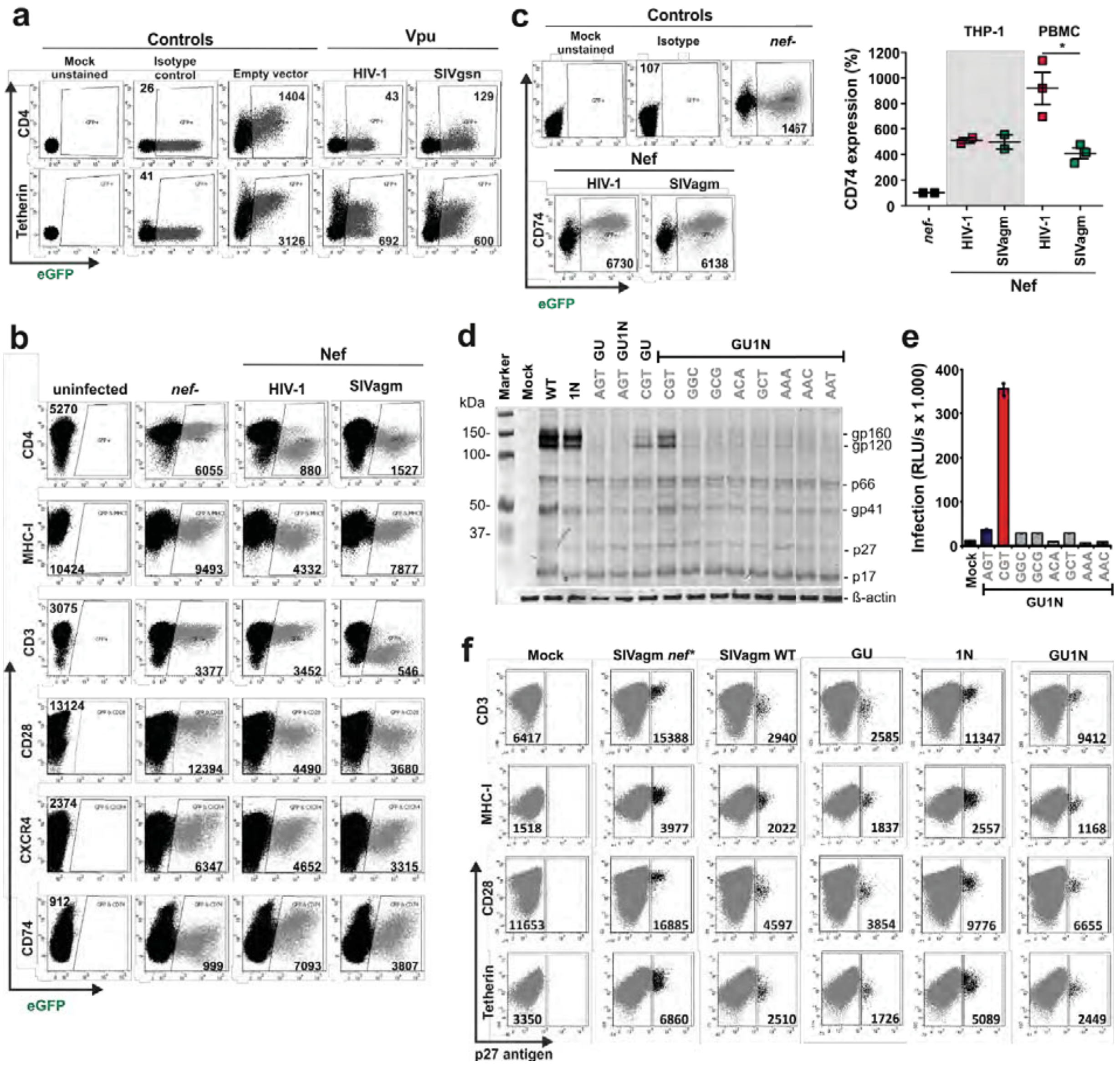

Supplementary Figure 1. Functional characterization and SIVagm proviral expression of the SIVgsn Vpu and HIV-1 NA7 Nef proteins. (**a**) Modulation of CD4 and AGM tetherin by HIV-1 WITO and SIVgsn71 Vpu. FACS analysis of HEK293T cells cotransfected with CD4 or AGM tetherin expression vectors and pCG plasmids expressing eGFP alone or together with the indicated *vpu* allele. (**b**) Human PBMCs or CD4+ T cells were stimulated with CD3/CD28 beads for three days, transduced with VSV-G pseudotyped *vpu*- and *env*-defective HIV-1 NL4-3 IRES eGFP constructs containing the indicated *nef* alleles and examined by flow cytometric analysis 2 days later. (**c**) Upmodulation of CD74 cell surface expression by HIV-1 NA7 and SIVagm Sab Nef proteins. THP-1 cells or human PBMCs were transduced and analyzed by flow cytometry for CD74 expression as described in panel b. Each symbol represents the result obtained in one independent experiment. (**d**) Western blot analysis of cellular extracts of HEK293T cells transfected with the indicated mutant SIVagm *vpu* Kozak variants. (**e**) Infectious virus production by HEK293T cells transfected with the indicated SIVagm GU1N constructs. Shown are average values (±SEM) of triplicate infections. (**f**) Modulation of CD3, MHC-I, CD28 and AGM tetherin in AGM PBMCs. The cells were stimulated with PHA for three days and then spinfected with the different SIVagm IMC. Surface expression by flow cytometric analysis was performed 2 days later.

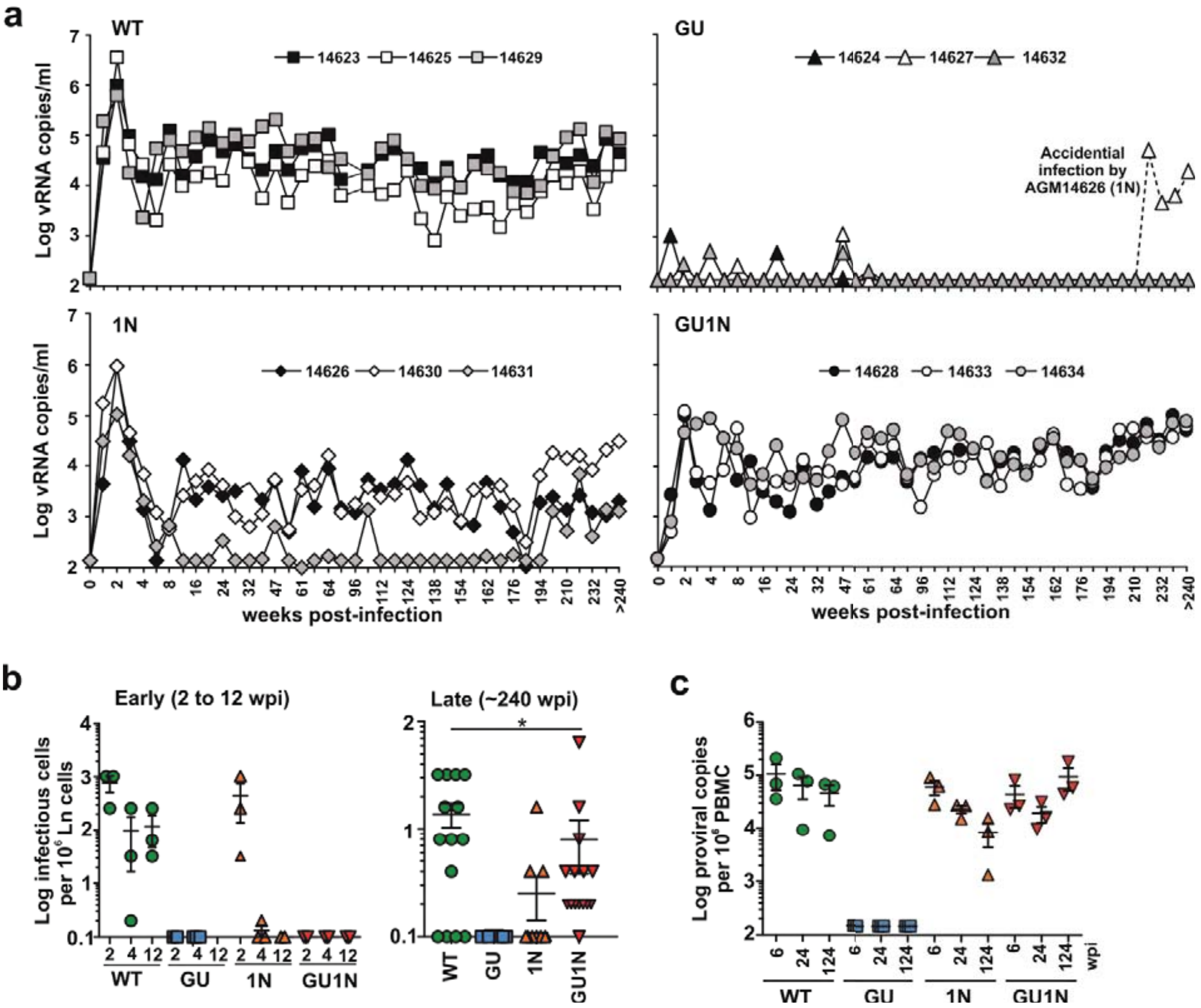

Supplementary Figure 2. Replication of chimeric SIVagm constructs *in vivo.* (**a**) Viral RNA copy numbers in AGMs infected with the four SIVagm constructs. (**b**) Cell-associated viral loads in lymph nodes (Ln) at the indicated time points. 12 wpi ewas not determined for the GU group (**c**) Proviral copy numbers in blood-derived PBMCs at later time points (>170 wpi). Panel b and c show the mean ± SEM.

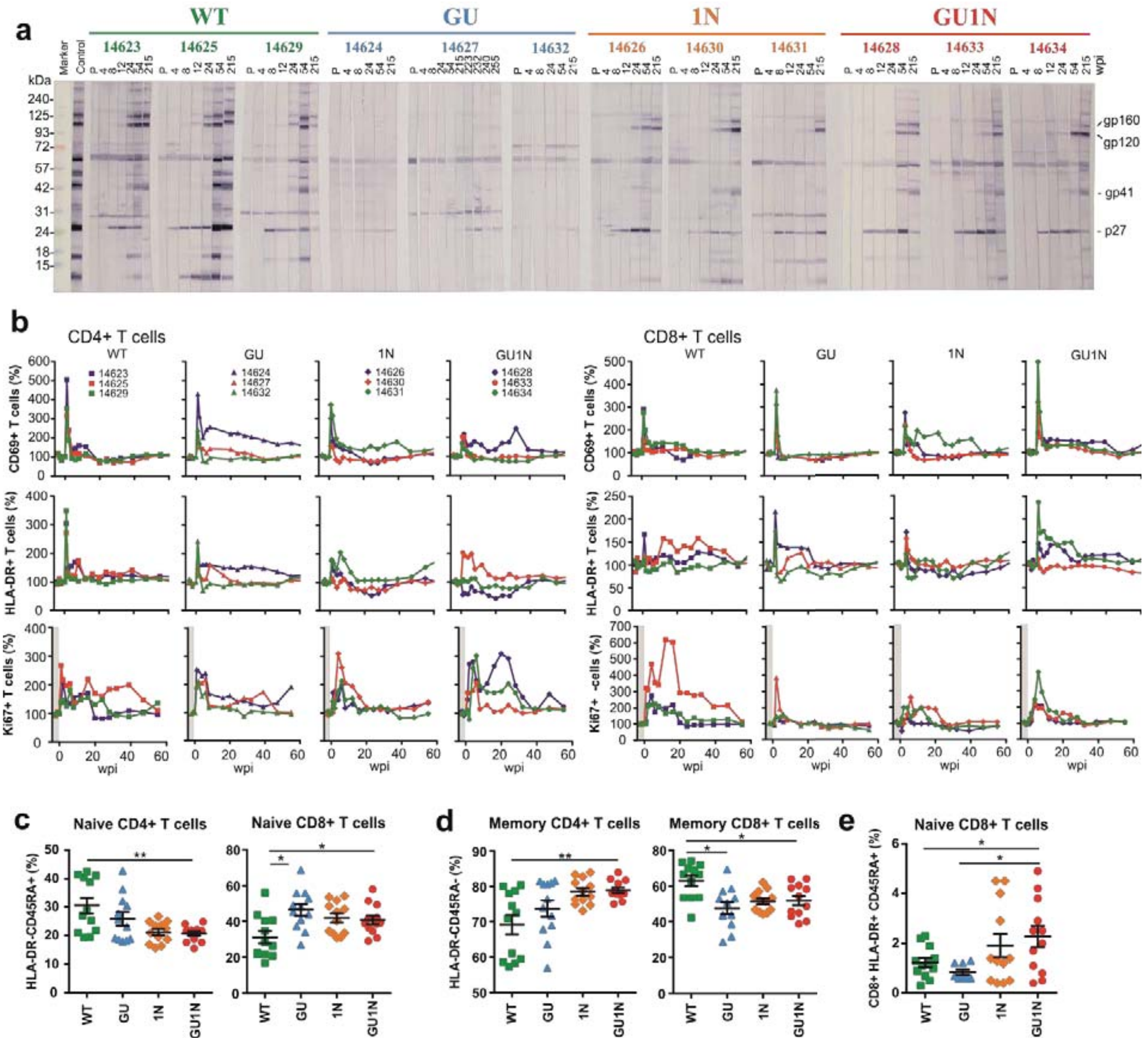

Supplementary Figure 3. Humoral immune response and cellular activation in WT and chimeric SIVagm infection. (**a**) Humoral immune response to SIVagm as determined by Western immunoblot. Viral proteins were separated by SDS-PAGE, transferred to a nitrocellulose membrane, and subjected to immunoblot with sera obtained from the infected AGMs at the indicated weeks post-infection (wpi). P, preserum; gp, glycoprotein. (**b**) Changes in the numbers of activated (CD69+ and HLA-DR+) and proliferating (Ki67+) CD4+ and CD8+ T cells compared to the average numbers detected prior to infection (100%). (**c-e**) Frequencies of (c) naïve, (c) memory and (e) activated naïve T cell subsets at later time points (≥170 wpi). Shown are individual data points with the mean ± SEM.

Figure S4a

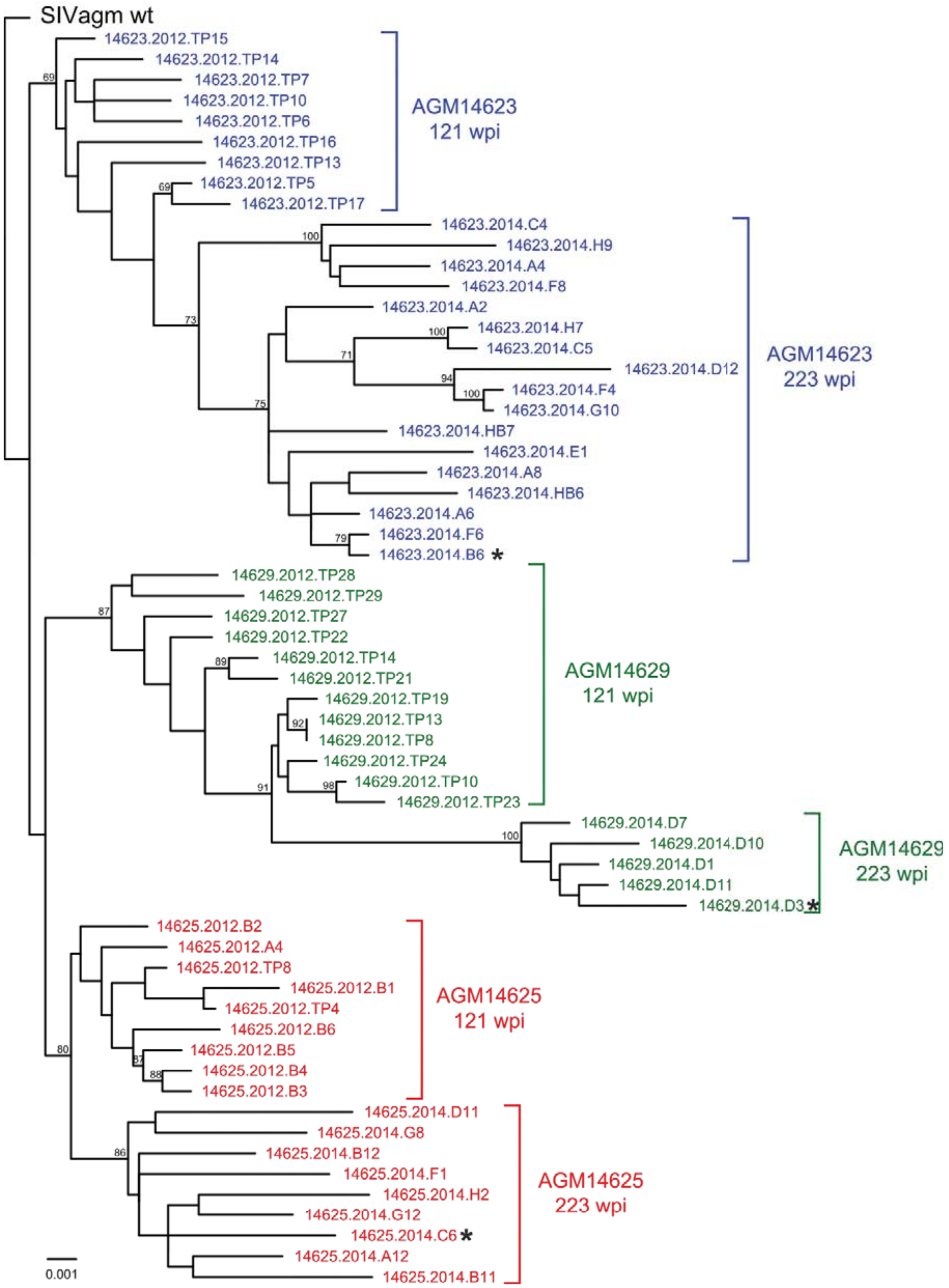
SIVagm wt
14623.2012.TP15
14623.2012.TP14
14623.2012.TP7
14623.2012.TP10
14623.2012.TP6
14623.2012.TP16
14623.2012.TP13
14623.2012.TP5
14623.2012.TP17
AGM14623
121 wpi
14623.2014.C4
14623.2014.H9
14623.2014.A4
14623.2014.F8
14623.2014.A2
14623.2014.H7
14623.2014.C5
14623.2014.D12
14623.2014.F4
14623.2014.G10
14623.2014.HB7
14623.2014.E1
14623.2014.A8
14623.2014.HB6
14623.2014.A6
14623.2014.F6
14623.2014.B6 *
AGM14623
223 wpi
14629.2012.TP28
14629.2012.TP29
14629.2012.TP27
14629.2012.TP22
14629.2012.TP14
14629.2012.TP21
14629.2012.TP19
14629.2012.TP13
14629.2012.TP8
14629.2012.TP24
14629.2012.TP10
14629.2012.TP23
AGM14629
121 wpi
14629.2014.D7
14629.2014.D10
14629.2014.D1
14629.2014.D11
14629.2014.D3 *
AGM14629
223 wpi
14625.2012.B2
14625.2012.A4
14625.2012.TP8
14625.2012.B1
14625.2012.TP4
14625.2012.B6
14625.2012.B5
14625.2012.B4
14625.2012.B3
AGM14625
121 wpi
14625.2014.D11
14625.2014.G8
14625.2014.B12
14625.2014.F1
14625.2014.H2
14625.2014.G12
14625.2014.C6 *
14625.2014.A12
14625.2014.B11
AGM14625
223 wpi
0.001

Figure S4b

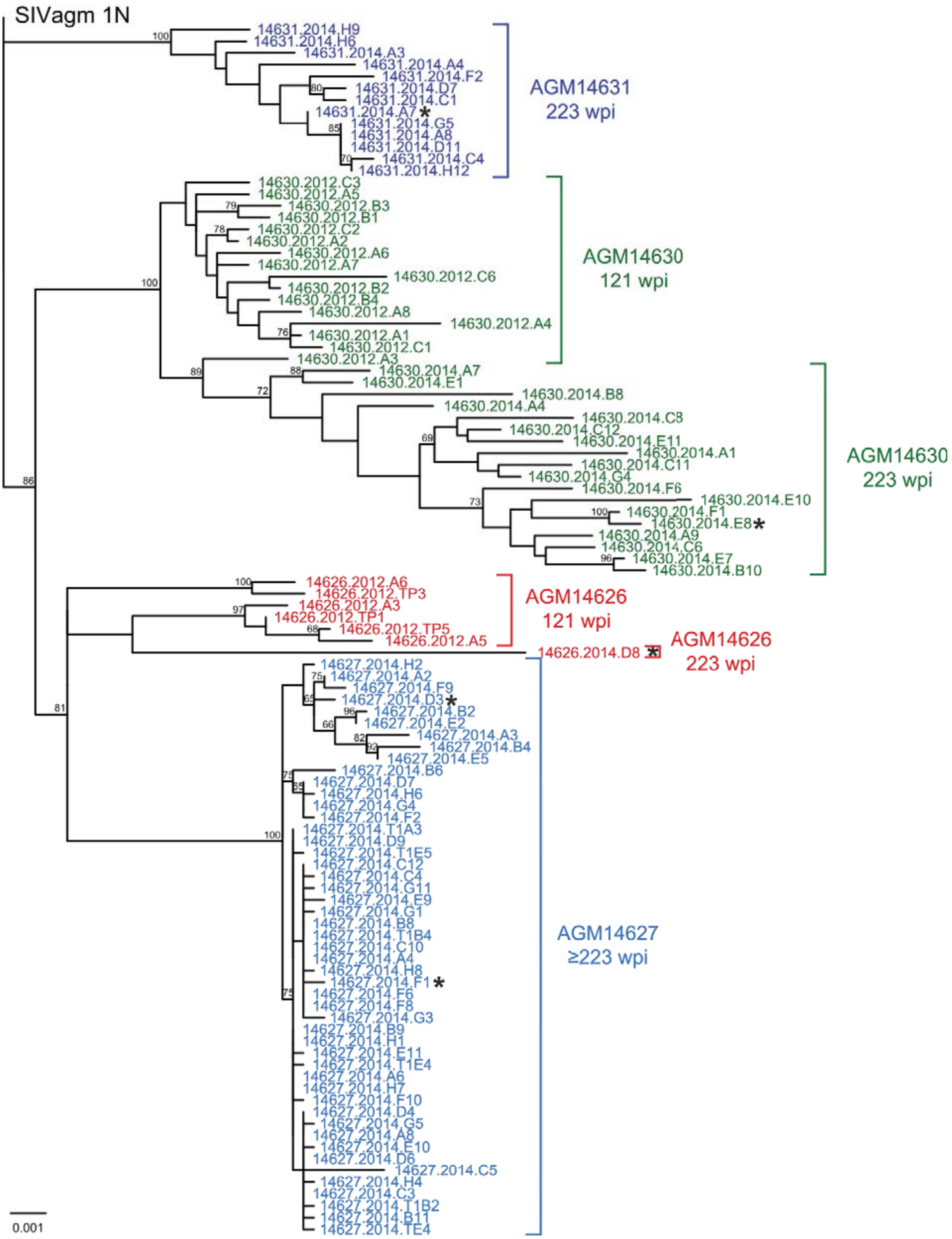
SIVagm 1N
14631.2014.H9
14631.2014.H6
14631.2014.A3
14631.2014.A4
14631.2014.F2
14631.2014.D7
14631.2014.C1
14631.2014.A7 *
14631.2014.G5
14631.2014.A8
14631.2014.D11
14631.2014.C4
14631.2014.H12
AGM14631
223 wpi
14630.2012.C3
14630.2012.A5
14630.2012.B3
14630.2012.B1
14630.2012.C2
14630.2012.A2
14630.2012.A6
14630.2012.A7
14630.2012.C6
14630.2012.B2
14630.2012.B4
14630.2012.A8
14630.2012.A4
14630.2012.A1
14630.2012.C1
14630.2012.A3
AGM14630
121 wpi
14630.2014.A7
14630.2014.E1
14630.2014.B8
14630.2014.A4
14630.2014.C8
14630.2014.C12
14630.2014.E11
14630.2014.A1
14630.2014.C11
14630.2014.G4
14630.2014.F6
14630.2014.E10
14630.2014.F1
14630.2014.E8 *
14630.2014.A9
14630.2014.C6
14630.2014.E7
14630.2014.B10
AGM14630
223 wpi
14626.2012.A6
14626.2012.TP3
14626.2012.A3
14626.2012.TP1
14626.2012.TP5
14626.2012.A5
AGM14626
121 wpi
14626.2014.D8 *
AGM14626
223 wpi
14627.2014.H2
14627.2014.A2
14627.2014.F9
14627.2014.D3 *
14627.2014.B2
14627.2014.E2
14627.2014.A3
14627.2014.B4
14627.2014.E5
14627.2014.B6
14627.2014.D7
14627.2014.H6
14627.2014.G4
14627.2014.F2
14627.2014.T1A3
14627.2014.D9
14627.2014.T1E5
14627.2014.C12
14627.2014.C4
14627.2014.G11
14627.2014.E9
14627.2014.G1
14627.2014.B8
14627.2014.T1B4
14627.2014.C10
14627.2014.A4
14627.2014.H8
14627.2014.F1 *
14627.2014.F6
14627.2014.F8
14627.2014.G3
14627.2014.B9
14627.2014.H1
14627.2014.E11
14627.2014.T1E4
14627.2014.A6
14627.2014.H7
14627.2014.F10
14627.2014.D4
14627.2014.G5
14627.2014.A8
14627.2014.E10
14627.2014.D6
14627.2014.C5
14627.2014.H4
14627.2014.C3
14627.2014.T1B2
14627.2014.B11
14627.2014.TE4
AGM14627
≥223 wpi
0.001

Figure S4c

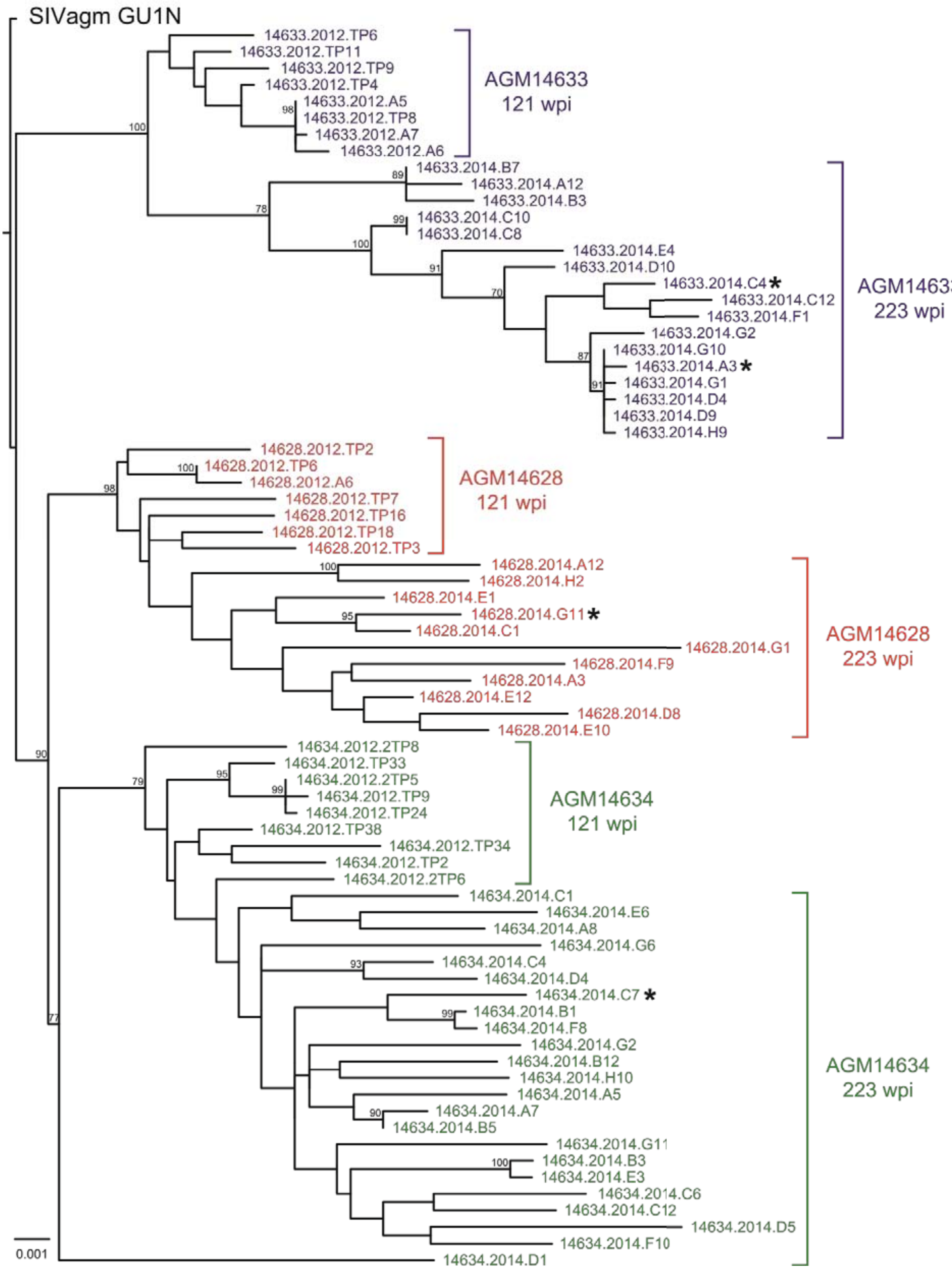
SIVagm GU1N
14633.2012.TP6
14633.2012.TP11
14633.2012.TP9
14633.2012.TP4
14633.2012.A5
14633.2012.TP8
14633.2012.A7
14633.2012.A6
AGM14633
121 wpi
14633.2014.B7
14633.2014.A12
14633.2014.B3
14633.2014.C10
14633.2014.C8
14633.2014.E4
14633.2014.D10
14633.2014.C4 *
14633.2014.C12
14633.2014.F1
14633.2014.G2
14633.2014.G10
14633.2014.A3 *
14633.2014.G1
14633.2014.D4
14633.2014.D9
14633.2014.H9
AGM14633
223 wpi
14628.2012.TP2
14628.2012.TP6
14628.2012.A6
14628.2012.TP7
14628.2012.TP16
14628.2012.TP18
14628.2012.TP3
AGM14628
121 wpi
14628.2014.A12
14628.2014.H2
14628.2014.E1
14628.2014.G11 *
14628.2014.C1
14628.2014.G1
14628.2014.F9
14628.2014.A3
14628.2014.E12
14628.2014.D8
14628.2014.E10
AGM14628
223 wpi
14634.2012.2TP8
14634.2012.TP33
14634.2012.2TP5
14634.2012.TP9
14634.2012.TP24
14634.2012.TP38
14634.2012.TP34
14634.2012.TP2
14634.2012.2TP6
AGM14634
121 wpi
14634.2014.C1
14634.2014.E6
14634.2014.A8
14634.2014.G6
14634.2014.C4
14634.2014.D4
14634.2014.C7 *
14634.2014.B1
14634.2014.F8
14634.2014.G2
14634.2014.B12
14634.2014.H10
14634.2014.A5
14634.2014.A7
14634.2014.B5
14634.2014.G11
14634.2014.B3
14634.2014.E3
14634.2014.C6
14634.2014.C12
14634.2014.D5
14634.2014.F10
14634.2014.D1
AGM14634
223 wpi
0.001

Supplementary Figure 4. Phylogenetic relationship of SIVagm sequences. (**a-c**) Phylogenetic analyses of sequences derived from the (a) WT, (b) 1N and (c) GU1N groups of infected AGMs. Panel b also includes sequences derived from the GU exposed animal 14627 that showed an unusual increase in plasma vRNA loads after >200 wpi. Maximum-likelihood phylograms were inferred using PhyML (Guindon et al. 2010) based on evolutionary substitution models favored by jModeltest (Daribba et al. 2012) for 3' half SGA sequences derived from the indicated animals at 121 or 223 wpi. Numbers at nodes are percent bootstrap support (only values >65% are shown) and asterisks indicate alleles selected for functional anaylsis of Vpu and/or Nef.

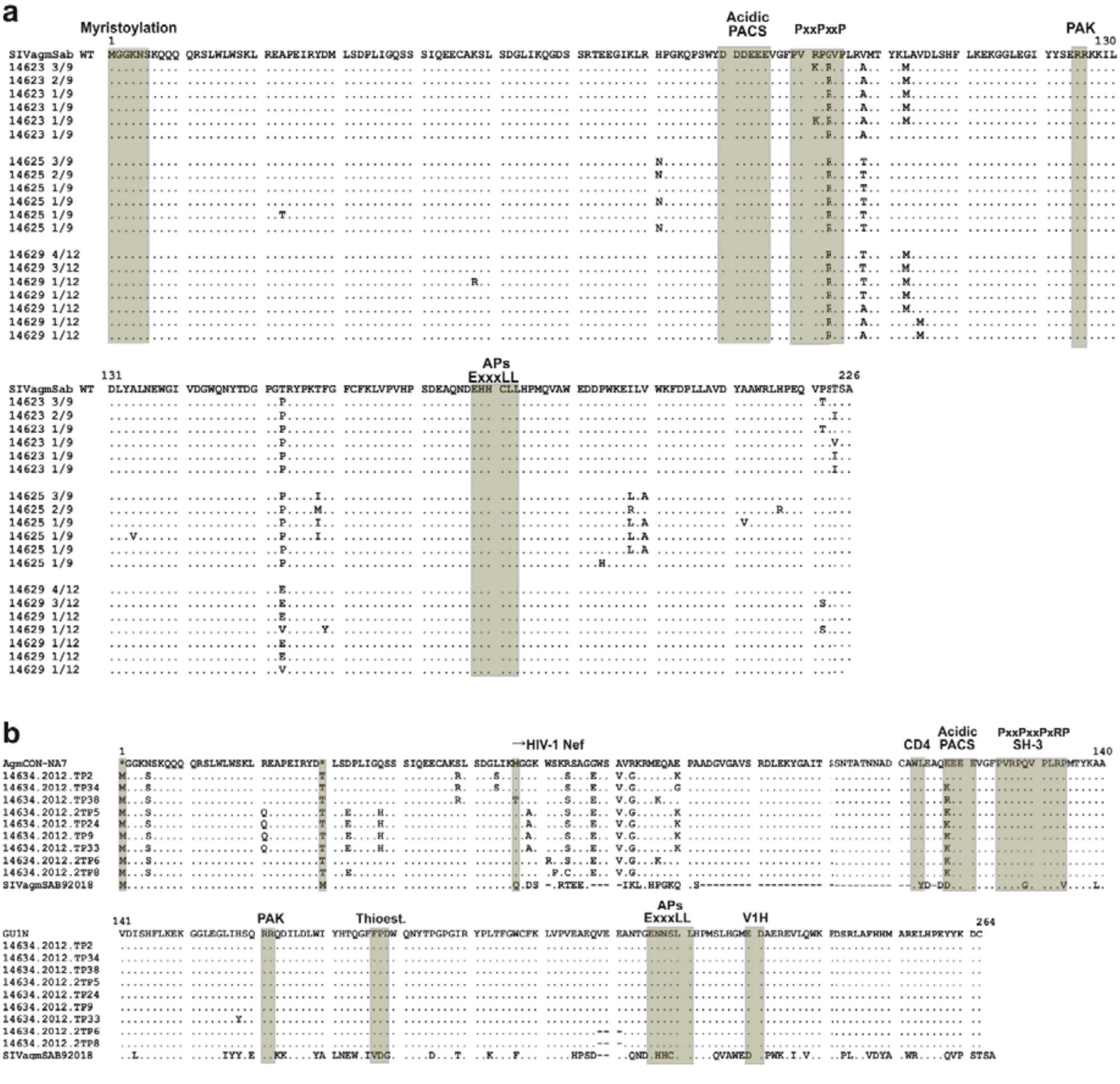

Supplementary Figure 5. Alignment of Nef sequences derived from WT infected AGMs and AGM14634. (**a**) The WT SIVagmSab Nef sequence is shown on top and several known functional domains are indicated. The numbers represent the frequency of the respective sequence and the total number of *nef* alleles analyzed. (**b**) Alignment of Nef-fusions detected in AGM14634.

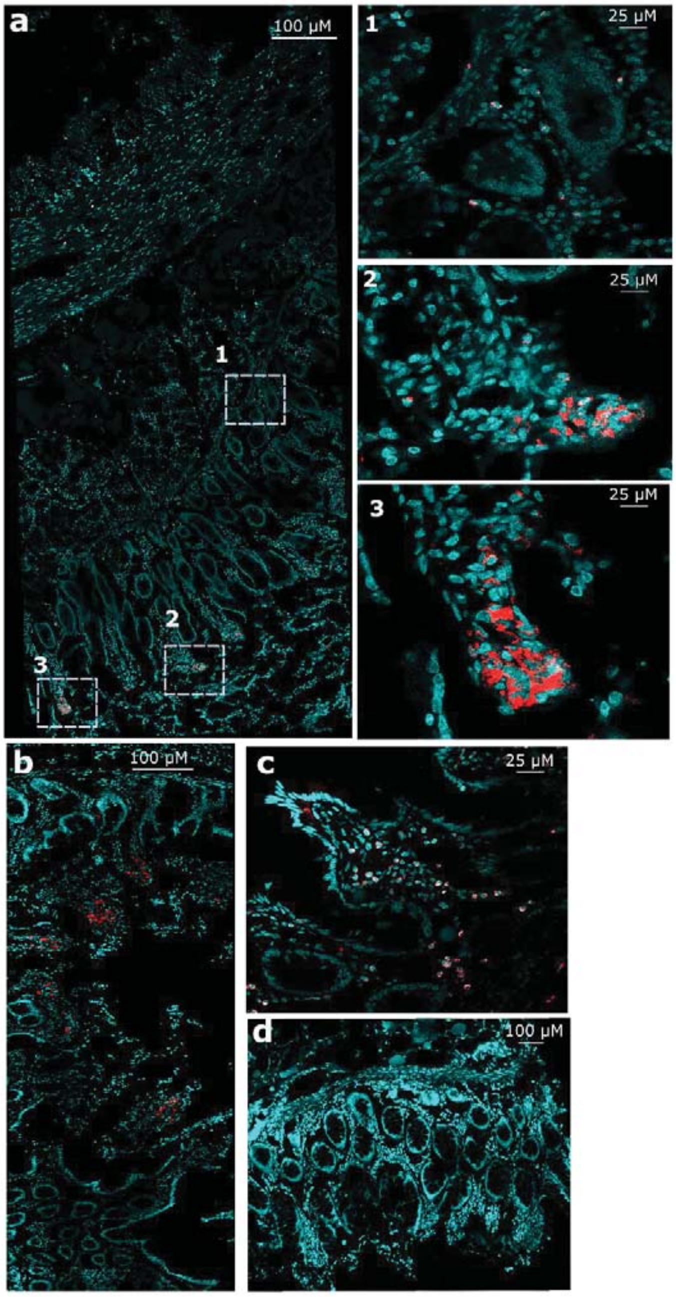

Supplementary Figure 6. Distribution of SIVagm GU in gut during the chronic phase of infection. Confocal images of SIV-infected cells (red), nucleus (blue) in duodenum (**a**) , ileum (**b**) and jejunum (**c**) are shown. Representative SIV-specific in situ hybridization during chronic infection, demonstrating productively infected cells in duodenum, ileum and jejunum. The enlargements display representative examples of the shape of the SIV RNA positive cells. Duodenum sections from an uninfected monkey were used as control to demonstrate the specificity of the SIV probes. Pictures were obtained using a Leica SP8 confocal microscope and processed with ImageJ software.

Figure 4b

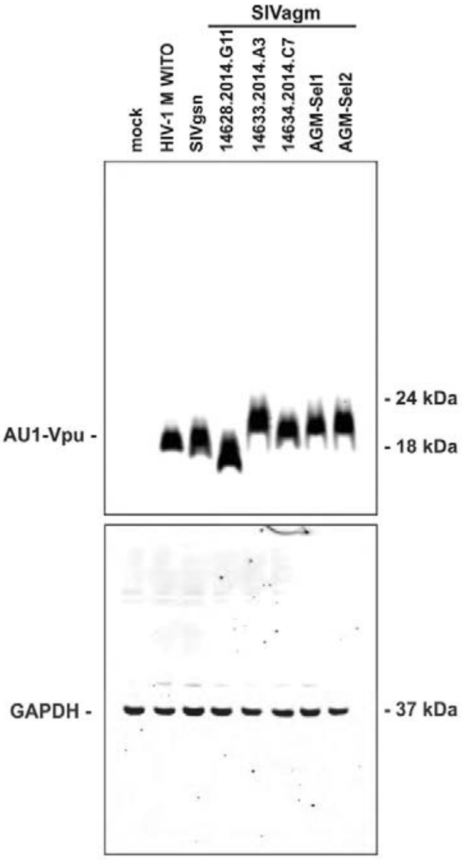

Figure 5b

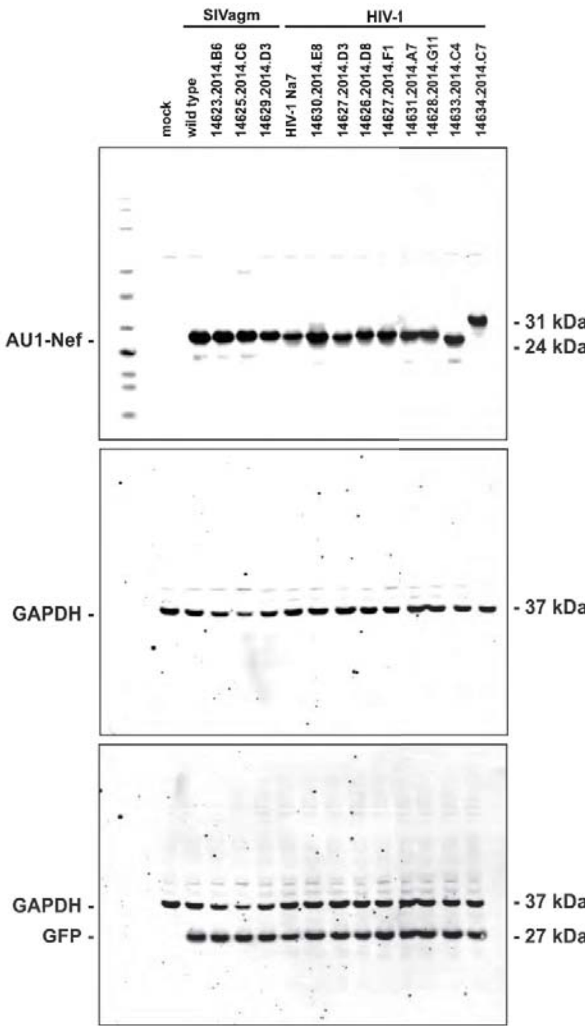

Supplementary Figure 7. Uncropped scans of the Western blots shown in the indicated figures.
